# Radiomics based on diffusion tensor imaging and 3D T1-weighted MRI for essential tremor diagnosis

**DOI:** 10.3389/fneur.2024.1460041

**Published:** 2024-08-27

**Authors:** Bintao Xu, Li Tao, Honge Gui, Pan Xiao, Xiaole Zhao, Hongyu Wang, Huiyue Chen, Hansheng Wang, Fajin Lv, Tianyou Luo, Oumei Cheng, Jing Luo, Yun Man, Zheng Xiao, Weidong Fang

**Affiliations:** ^1^Department of Radiology, The First Affiliated Hospital of Chongqing Medical University, Chongqing, China; ^2^Department of Neurology, The First Affiliated Hospital of Chongqing Medical University, Chongqing, China

**Keywords:** essential tremor, machine learning, radiomics, diffusion tensor imaging, 3D T1-weighted MRI

## Abstract

**Background:**

Due to the absence of biomarkers, the misdiagnosis of essential tremor (ET) with other tremor diseases and enhanced physiologic tremor is very common in practice. Combined radiomics based on diffusion tensor imaging (DTI) and three-dimensional T1-weighted imaging (3D-T1) with machine learning (ML) give a most promising way to identify essential tremor (ET) at the individual level and further reveal the potential imaging biomarkers.

**Methods:**

Radiomics features were extracted from 3D-T1 and DTI in 103 ET patients and 103 age-and sex-matched healthy controls (HCs). After data dimensionality reduction and feature selection, five classifiers, including the support vector machine (SVM), random forest (RF), logistic regression (LR), extreme gradient boosting (XGBoost) and multi-layer perceptron (MLP), were adopted to discriminate ET from HCs. The mean values of the area under the curve (mAUC) and accuracy were used to assess the model’s performance. Furthermore, a correlation analysis was conducted between the most discriminative features and clinical tremor characteristics.

**Results:**

All classifiers achieved good classification performance (with mAUC at 0.987, 0.984, 0.984, 0.988 and 0.981 in the test set, respectively). The most powerful discriminative features mainly located in the cerebella-thalamo-cortical (CTC) and visual pathway. Furthermore, correlation analysis revealed that some radiomics features were significantly related to the clinical tremor characteristics in ET patients.

**Conclusion:**

These results demonstrated that combining radiomics with ML algorithms could not only achieve high classification accuracy for identifying ET but also help us to reveal the potential brain microstructure pathogenesis in ET patients.

## Introduction

Essential tremor (ET) brings about a considerable global health burden, affecting approximately 1% of the world’s population (1). Recently, the International Parkinson and Movement Disorder Society redefined ET as a bilateral isolated upper limb action tremor syndrome lasting for a minimum of 3 years, and ET with other soft neurological signs such as impaired tandem gait, questionable dystonic posturing and memory impairment were referred to as ET-plus (2). The design of a “pure” ET subtype with a more precise and narrow definition seemed to make the diagnosis of ET easier in clinical settings. However, due to the absence of pathological, genetic and neuroimaging biomarkers, the misdiagnosis of ET with Parkinson’s disease (PD), dystonia and enhanced physiologic tremor is very common in practice (3, 4). Therefore, establishing biomarkers of ET, especially imaging markers, is an extremely urgent task at present.

Diffusion tensor imaging (DTI) and high-resolution three-dimensional T1-weighted imaging (3D-T1) as non-invasive and *in vivo* magnetic resonance imaging (MRI) sequences have been widely used to measure brain microstructural changes and further construct the potential imaging markers in a lot of neurodegenerative diseases and movement disorders, such as Alzheimer’s disease, PD, dystonia, and multiple system atrophy (5–7). Recently, using 3D-T1 and DTI analysis, very few studies gained some variable and inconsistent findings, and some of these studies supported that the dentato-rubro-thalamic tract and its structure connectivity brain areas were associated with ET patients (8, 9). However, most of these studies were traditional mass univariate analyses, and they could not be used to predict ET patients at an individual level. Furthermore, these 3D-T1 and DTI analysis methods limited to traditional metrics such as the average value of gray matter (GM) volumes or thickness, fractional anisotropy (FA), radial diffusivity (RD), axial diffusivity (AD) and mean diffusivity (MD), and actually, these images not only provided information on different aspects of these microstructures but also contained vast numbers of quantitative information, such as radiomics features. Radiomics analysis can abstract vast quantitative features, including first-order statistical information from DTI and 3D-T1, and then these features are inputted for machine learning (ML) algorithms (10). ML builds optimal models by learning and training from massive input data and then applies the model to new data to predict and analyze diseases based on a single-subject level (11). To our knowledge, up to now, no studies have combined radiomic analysis based on DTI and 3D-T1 to identify ET patients from HCs.

Moreover, it is crucial to understand the potential clinical implications of these imaging markers. Radiomic features extracted from imaging data can potentially correlate with clinical variables, which may offer deeper insights into the pathology and progression of ET. Establishing these correlations can not only aid in the accurate diagnosis of ET but also help in monitoring disease progression and treatment response.

Hence, we aimed to explore whether combined radiomic analysis of DTI and 3D-T1 with multiple ML algorithms could be used to effectively distinguish ET patients from HCs and to evaluate the radiomics correlates with clinical variables of interest for ET pathology. We also expected that our proposed method would not only reveal the brain microstructural changes but also further help to understand brain microstructural pathogenesis in ET.

## Materials and methods

### Participants

This study was approved by the Ethics Committee of the First Affiliated Hospital of Chongqing Medical University (Chongqing, China) in accordance with the Helsinki Declaration ethical principles. All patients fulfilled the following criteria: (1) the ET diagnosis met the 2018 Movement Disorders Consensus Criteria (2), and all patients had annual follow-ups through the outpatient department or telephone; (2) the patients had an onset age between 18 to 55 years, and patients with earlier or later onset were not included; (3) the patients were without any apparent cognitive impairment (Mini-Mental State Examination (MMSE) scores >24); (4) the patients were without PD, dystonia, psychogenic tremor, thyroid disease, stroke, epilepsy, head injury or any other neurological dysfunction; (5) the patients were without other neurological soft signs, such as dystonia, ataxia, parkinsonism, rest tremor or non-motor symptoms, that is ET-plus patients did not include in this study. In addition, none of the HCs reported having first-or second-degree relatives with ET, and all subjects met DTI image quality control standards. Ultimately, 206 participants were enrolled, including 103 ET patients and 103 age-, sex-, and education-matched HCs, all right-handed.

Tremor severity was assessed with the Fahn-Tolosa-Marin Tremor Rating Scale (TRS). Meanwhile, to consider a ceiling effect for severe tremor while tremor amplitude >4 cm for the TRS scale, the Essential Tremor Rating Assessment Scale (TETRAS) was also adopted to assess tremor severity. The Hamilton Anxiety Rating Scale (HARS-14) and the 17-item Hamilton Depression Rating Scale (HDRS-17) were adopted to assess the anxiety and depression severity of all participants. The MMSE was used to briefly assess cognitive function and screen for dementia.

### MRI acquisition and data preprocessing

3D-T1, DTI and T2-FLAIR images were acquired using a GE Signa Hdxt 3-T scanner (General Electric Medical Systems, Milwaukee, WI, United States); for detailed parameters, see Supplementary material S1. Data preprocessing was conducted using the VBM implemented in SPM12 software^1^ and PANDA toolbox version 2.2,^2^ and detailed data preprocessing steps are provided in Supplementary material S2.

### Radiomics feature extraction

Previous studies have demonstrated that the brain microstructural changes were not only limited to white matter (WM) fiber tracts but also extended to gray matter (GM) areas. To capture these changes, the automated anatomical labeling 3 (AAL3) (12) and Johns Hopkins University (JHU) (13) tractography atlases were utilized. The FA, AD, RD, and MD maps of DTI were partitioned into 214 volumes of interest (VOIs), which included 164 regions defined by AAL3 and 50 regions defined by JHU-ICBM. Similarly, the GM maps of 3D-T1 were partitioned into 164 VOIs by AAL3, while the WM maps of 3D-T1 were partitioned into 50 VOIs by JHU-ICBM. The open-source Python package, pyradiomics, was employed to extract 15 first-order features, including the mean, median, maximum, range, variance, skewness, kurtosis, 10th percentile, 90th percentile, inter-quartile range, mean absolute deviation, robust mean absolute deviation, root mean squared, energy and total energy. These features were used to describe the voxel intensity distribution within the image mask (detailed information about extracted features is reported in Supplementary Table S1) (14). After the above process, 12,300 (164 × 15 × 5) GM features and 3,750 (50 × 15 × 5) WM features were obtained for every subject. GM features were sourced from GM regions defined by AAL3 in the FA, AD, RD, and MD maps of DTI, as well as the GM maps of 3D-T1. WM features were sourced from WM regions defined by JHU-ICBM in the FA, AD, RD, and MD maps of DTI, as well as the WM maps of 3D-T1.

### Feature selection

The machine-learning analysis was performed by using a scikit-learn open-source package^3^ in Python. Due to the curse-of-dimensionality or small-n-large-p problem, a total of 16,050 features greatly exceeded the sample size, while most features were redundant and irrelevant (15). Therefore, dimensionality reduction and feature selection were necessary steps to obtain the most important features and improve the accuracy of the model. Before the feature selection, the dataset was partitioned into training and testing sets in the ratio of 7:3, and a Z-score standardization was performed, respectively, to keep the data in sets mutually independent. Then, dimensionality reduction and feature selection were conducted in the training set in three steps. First, we conducted a two-sample t-test to assess the statistical significance of the relationship between each feature and the target variable. Features with a *p*-value below 0.05 were deemed statistically significant. Next, we employed the mutual information method to filter out features that showed a low correlation with the target variable, setting a threshold of 0.05. Lastly, we utilized the absolute shrinkage and selection operator (LASSO) algorithm in the feature selection process. LASSO is a regression method that addresses the issue of multicollinearity by shrinking the coefficients of less critical features toward zero, thereby effectively eliminating redundant features from the model. The key to LASSO’s effectiveness lies in its penalization parameter λ, a hyperparameter that controls the degree of regularization of the model. It was tuned under the criteria of minimal mean squared error (MSE) to construct the optimal subset of features via a 10-fold cross-validated grid-search approach, and the weight coefficients of each feature were calculated. The loss function of LASSO is as follows:


$$L=\overset{n}{\underset{i=1}{\sum }}{\left(y_{i}-\hat{y}i\right)}^{2}+\lambda \overset{P}{\underset{j=1}{\sum }}|\beta_{j}|$$

where $y_{i}$ are the observed values, $\hat{y}i$ are the predicted values, λ is the penalization parameter, $\beta_{j}$ are the coefficients of the features, n is the number of observations, and P is the number of features.

### Model construction and evaluation

In order to enhance the performance and generalization ability of our models, we employed nested loops to perform hyperparameter tuning and make full use of subject data. Initially, the entire dataset was split into a training set and a test set in a 7:3 ratio using stratified splitting, ensuring that the proportions of the two classes were balanced in both sets. The independent test set served as the outer loop for evaluating model performance, while the training set after dimensionality reduction and feature selection was used as the inner loop for 10-fold cross-validation and grid search to determine the optimal classifier parameters. In each fold of the inner loop, various hyperparameter combinations were attempted, and model scores were recorded, with the combination yielding the highest score selected as the optimal hyperparameters, which were then fitted to the entire training set and evaluated on the test set. We employed several common machine learning classifiers, including the support vector machine with radial basis function kernel (RBF-SVM) (16), random forest (RF) (17), logistic regression with the linear kernel (Linear-LR) (18), extreme gradient boosting (XGBoost) (19) and multi-layer perceptron (MLP) (20), to build models based on the preserved features from feature selection. Specifically, we searched for the optimal hyperparameters for RBF-SVM (penalty parameter C), Linear-LR (parameter C), RF (number of decision trees), XGBoost (number of decision trees, maximum depth, learning rate), and MLP (hidden layer size, activation function, optimizer) classifiers. To ensure unbiased classification estimates, the entire framework was repeated 100 times. The whole procedure for the nested loop is illustrated in Supplementary Figure S1.

The model’s performance was evaluated using an independent test set in the outer loop, ensuring a more representative evaluation of the model’s ability to generalize. To gage model performance, we computed metrics, including mean accuracy (mACC), mean balanced accuracy (mBACC), mean sensitivity (mSN), and mean specificity (mSP). We also constructed the mean receiver operating characteristic (mROC) curve and calculated the mean area under the curve (mAUC) to gauge the models’ classification performance and diagnostic accuracy. The model that achieved the highest mAUC value was considered the best-performing model. To compare the different classification algorithms, we utilized the Friedman test followed by the Wilcoxon signed-rank test for pairwise comparisons when significant differences were identified. To correct for multiple comparisons, we applied the Bonferroni method, considering an adjusted alpha (α) level of <0.05 as statistically significant. The formulae are as follows:

$\mathrm{Sensitivity}=TP/\left(TP+FN\right)$

$\mathrm{Specificity}=TN/\left(TN+FP\right)$

$\mathrm{Balanced}\;\mathrm{accuracy}=\left(\mathrm{Sensitivity}+\mathrm{Specificity}\right)/2$

where TP represents the number of positive samples correctly classified, TN represents the number of negative samples correctly classified, FP represents the number of negative samples incorrectly classified, and FN represents the number of positive samples incorrectly classified.

To assess the statistical significance of the classification model, we conducted permutation testing by randomly shuffling the labels of both patients and HCs. This process was iterated 1,000 times, and the entire framework was executed on each occasion. We then compared the obtained classification performance metrics with those generated using randomly reassigned labels and calculated the corresponding *p*-value (21). A p-value below the significance threshold of 0.05 indicates a robust classification performance, providing compelling evidence that the classifier effectively distinguishes between the two groups.

### Identification of discriminative features

Considering that the dataset was randomly divided into a 7:3 ratio and the entire process was repeated 100 times, each iteration resulted in slightly different compositions of training and testing sets. This inherent variability meant that different features might be selected during each iteration of the feature selection process. To ensure that the final subset of features is representative and robust, features that were selected in more than 60 iterations were deemed relevant for distinguishing between individuals with ET and HCs. This method helps mitigate the risk of overfitting to any particular random split of the data, enhancing the generalizability of our model. Moreover, features that appear consistently across numerous iterations are likely to capture fundamental patterns and relationships within the data, making them more reliable for distinguishing between ET and HCs. We then computed the average feature weights. The absolute value of the average feature weight indicates the feature’s contribution to the model’s classification performance. Features with larger average feature weights are considered more significant in terms of their impact on the model’s discriminative capability. The whole radiomics analysis workflow is illustrated in Supplementary Figure S2.

### Statistical analysis

We analyzed the demographic data and clinical characteristics of both groups using SPSS statistical software. Initially, we assessed the normality of continuous variables with the Kolmogorov–Smirnov test (K-S test). For normally distributed variables, we conducted a two-sample t-test, while for non-normally distributed variables, we employed the Mann–Whitney U test. To examine differences in qualitative data, such as gender, we used the chi-square test. A two-tailed *p*-value <0.05 was regarded as significant. Furthermore, we performed partial Pearson correlation analysis to explore potential relationships between the selected features and clinical tremor status, as indicated by scale scores. Meanwhile, the age, gender, education years, and scores of the MMSE, HARS-14, and HDRS-17 as covariates, applying Bonferroni multiple comparison correction (*p* < 0.05/10*(10–1)/2 = 0.001).

## Results

### Demographic and clinical characteristics

Demographic and clinical data for all participants are summarized in Table 1. There were no statistically significant differences between the ET group and HCS group in age, gender, education level, handedness, smoking status, HDRS-17, HDRS-14 scores, etc. (*p* > 0.05). However, there was a significant difference in MMSE scores between the two groups, with the ET group scoring lower than the HCS group (*p* = 0.0006).

**Table 1:** Demographic and clinical features of ET and HCs.

Measure	ET	HCs	Statistics	*p*-value
Demographic				
Sample size	103	103	NA	NA
Age (years)	48.06 ± 14.38	44.34 ± 13.79	T = 1.89	0.0596
Gender (M:F)	47:56	58:45	Z = −1.53	0.1261
Education (years)	12.53 ± 4.55	12.41 ± 4.65	T = 0.18	0.8559
Handedness (R/L)	103:0	103:0	*Z* = −0.00	1.0000
Cigarette smoker	47	58	*Z* = −1.53	0.1261
Clinical of tremor				
Tremor of onset (years)	35.68 ± 10.70	NA	NA	NA
Tremor of duration (years)	12.38 ± 8.31	NA	NA	NA
Positive family history		NA	NA	NA
Positive	37	NA	NA	NA
Negative	66	NA	NA	NA
Alcohol sensitivity		NA	NA	NA
Positive	37	NA	NA	NA
Negative	40	NA	NA	NA
NA	26	NA	NA	NA
Tremor medication		NA	NA	NA
Propranolol	30(43.33 ± 16.47 mg)	NA	NA	NA
Primidone	4 (200.00 ± 91.29 mg)	NA	NA	NA
Tremor symmetry		NA	NA	NA
R = L	93	NA	NA	NA
R < L	7	NA	NA	NA
R > L	3	NA	NA	NA
Tremor frequency	6.84 ± 2.13	NA	NA	NA
TRS-parts A&B	22.93 ± 7.91	NA	NA	NA
TRS-part C	12.57 ± 7.15	NA	NA	NA
TETRAS	21.09 ± 6.84	NA	NA	NA
TET-ADSL	12.72 ± 6.28	NA	NA	NA
Clinical of psychology and cognitive				
HDRS-17	2.05 ± 1.01	2.11 ± 1.32	T = −0.34	0.7326
HARS-14	2.75 ± 1.15	2.50 ± 1.84	T = 1.18	0.2408
MMSE	28.57 ± 1.30	29.18 ± 1.23	T = −3.45	0.0006

ET, essential tremor; HCs, healthy controls; HDRS-17, 17-item Hamilton Depression Rating Scale; MMSE, Mini-Mental State Examination; HARS-14, 14-item Hamilton Anxiety Rating Scale; TRS, Fahn-Tolosa-Marin Tremor Rating Scale; NA, not available.

### Discriminative features

**Figure 1:**
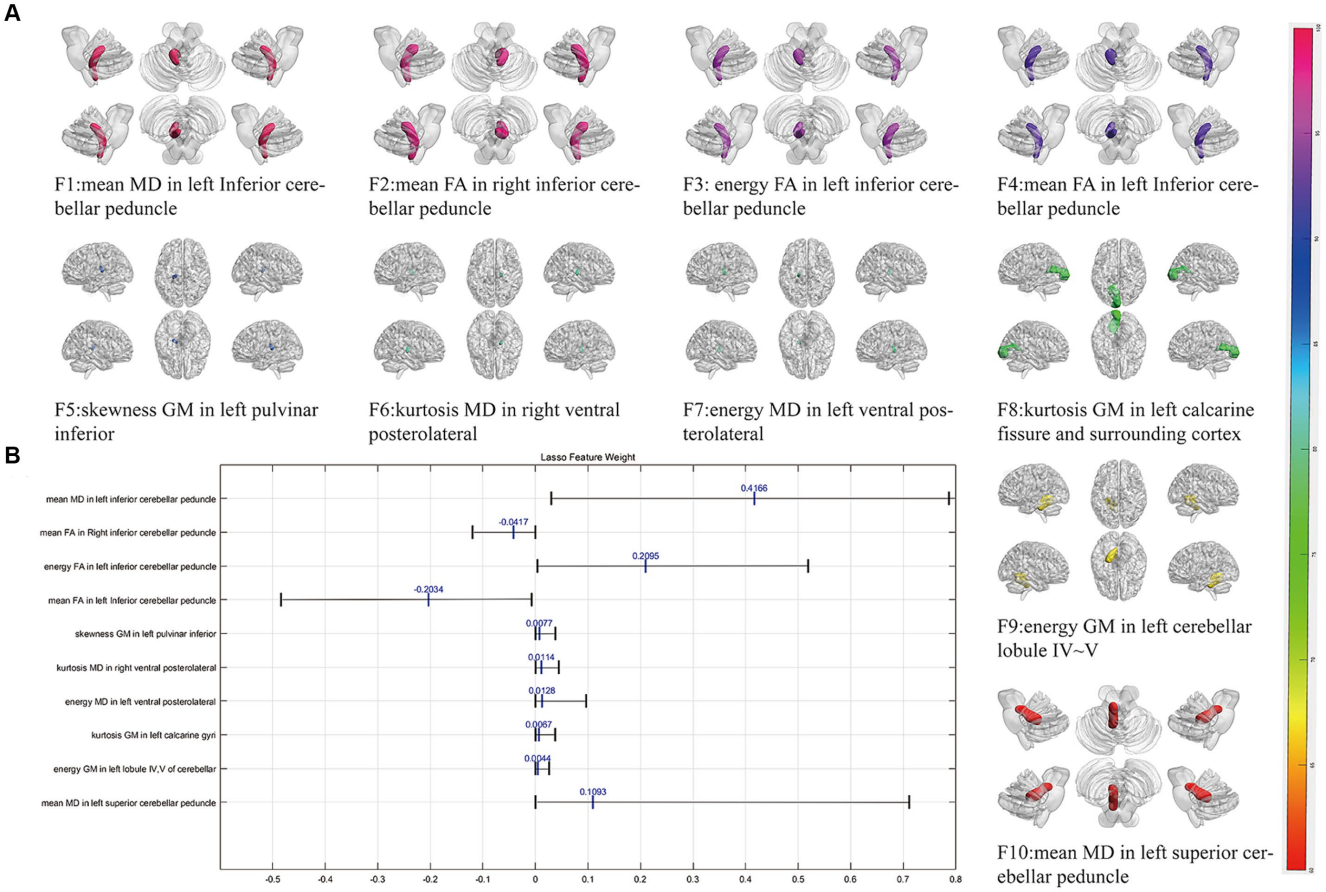
The selected most power discriminative features. **(A)** Showed the alignment diagram based on the coefficients in the LASSO analysis of the most discriminative features, with the black horizontal line segments representing the range of the coefficients, with the left end indicating the minimum value and the right end indicating the maximum value. The blue line represented the mean value of the coefficients. **(B)** Showed the most power discriminative features between ET and HCs groups and the color bar value represents the frequency of the features. FA, fractional anisotropy; MD, mean diffusivity; GM, gray matter.

Following three steps of feature reduction, an average of approximately 46 features were retained (range: 16 to 82) per round, with an average Lasso penalization parameter λ of 0.0052. Due to the complete random sampling of the training and test sets, the training set samples for feature selection were different, and the retained features varied in each round. With 100 repetitions of the entire framework, a total of 100 feature subsets containing different selected features were obtained. For the final distinguishing subset of features, we considered only those features that were selected in more than 60 iterations. Ultimately, 10 features met this criterion (Table 2; Figure 1): mean MD in left inferior cerebellar peduncle (ICP), mean FA in right inferior cerebellar peduncle (ICP), energy FA in left inferior cerebellar peduncle (ICP), mean FA in left inferior cerebellar peduncle (ICP), skewness GM in left pulvinar inferior(tPuL), kurtosis MD in right ventral posterolateral(tVPL), energy MD in left ventral posterolateral(tVPL), kurtosis GM in left calcarine fissure and surrounding cortex(CAL), energy GM in left cerebellar lobule IV ~ V(CER4_5) and mean MD in left superior cerebellar peduncle(SCP). The most frequent and highest-weighted feature was Mean in the FA map located in the left inferior cerebellar peduncle, occurring 100 times out of 100 rounds with an average weight of 0.416.

**Table 2:** The significant discriminative features between ET and HCs.

Brain region	Hemisphere	Image type	Statistics	Frequency
Inferior cerebellar peduncle	Left	Mean diffusivity	Mean	100
Inferior cerebellar peduncle	Right	Fractional anisotropy	Mean	99
Inferior cerebellar peduncle	Left	Fractional anisotropy	Energy	95
Inferior cerebellar peduncle	Left	Fractional anisotropy	Mean	92
Pulvinar inferior	Left	Gray matter	Skewness	87
Ventral posterolateral	Right	Mean diffusivity	Kurtosis	80
Ventral posterolateral	Left	Mean diffusivity	Energy	79
Calcarine fissure and surrounding cortex	Left	Gray matter	Kurtosis	77
lobule IV,V of cerebellar hemisphere	Left	Gray matter	Energy	67
Superior cerebellar peduncle	Left	Mean diffusivity	Mean	60

### Classification performance

**Figure 2:**
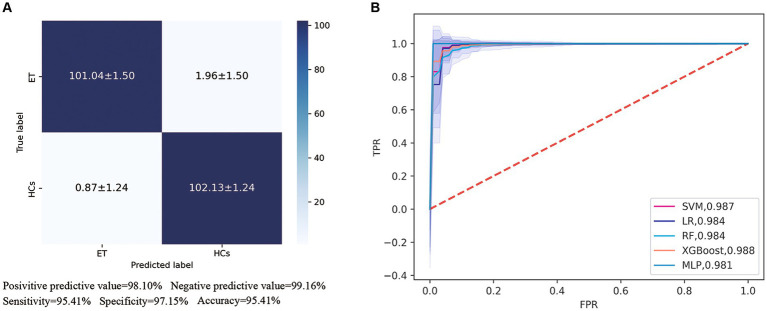
Receiver operating characteristic (ROC) curves and area under the curve (AUC) of five machine learning models. **(A)** Showed the confusion matrix of the best classifier-XGBoos based on 100 cycles. **(B)** Showed the ROC curves and AUC values of all classifiers on the test set. SVM, the support vector machine; RF, random forest; LR, logistic regression; XGBoost, extreme gradient boosting; MLP, multi-layer perceptron.

**Figure 3:**
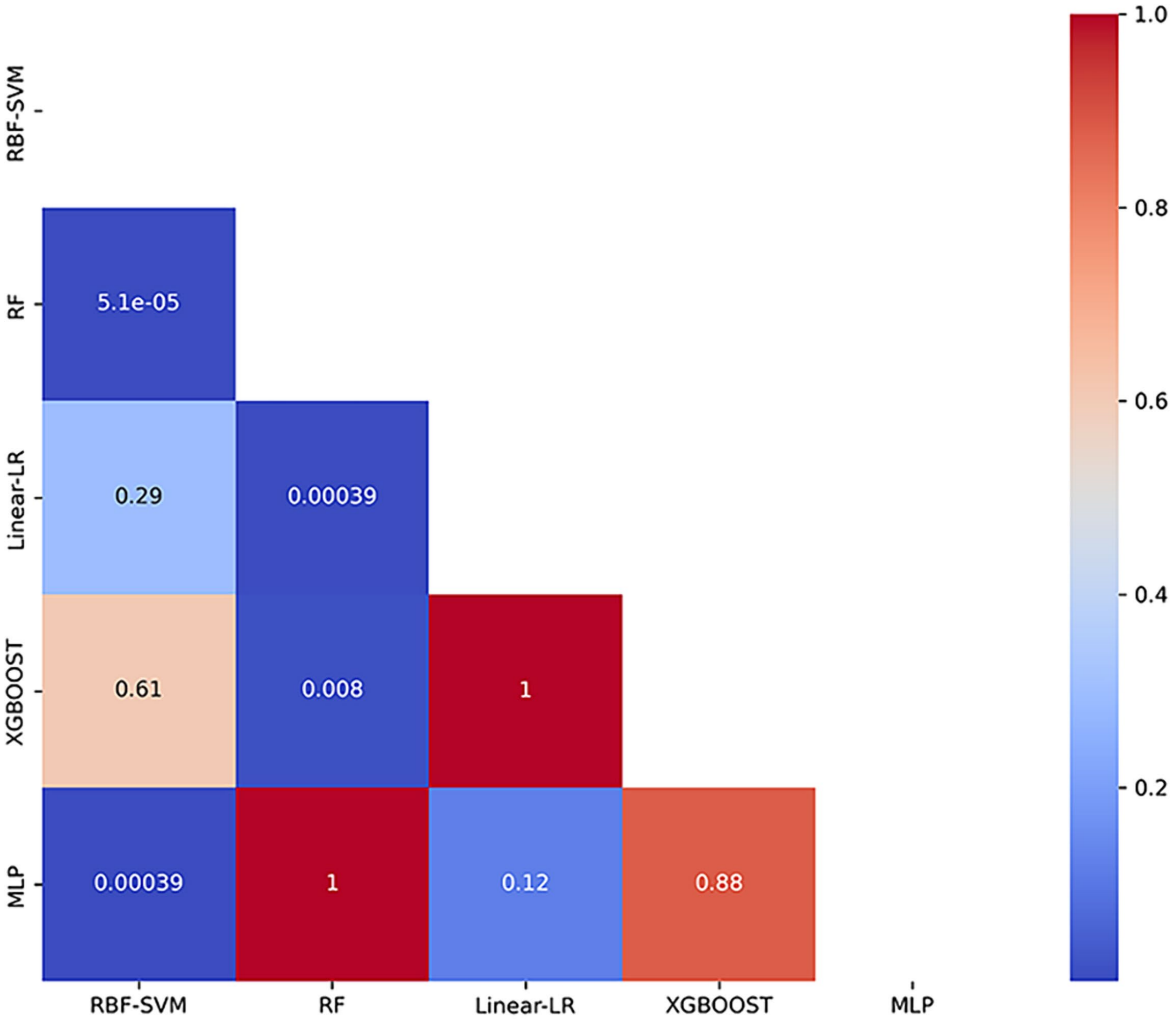
Heatmap of *p*-values from Wilcoxon Signed-Rank Test with Bonferroni Correction. The heatmap presents the *p*-values obtained from pairwise comparisons of classification models using the Wilcoxon signed-rank test, adjusted with the Bonferroni correction. RBF-SVM, the support vector machine with radial basis function kernel; RF, random forest; Linear-LR, logistic regression with the linear kernel; XGBoost, extreme gradient boosting; MLP, multi-layer perceptron.

In our automated classification framework, we employed five classifiers. To thoroughly evaluate the classification performance, we repeated the entire framework 100 times and assessed the model’s average performance across these 100 rounds. All classifiers achieved good classification performance with little overfitting (Figure 2 and Table 3). Evaluating a machine learning model’s performance on a test set is crucial, as it provides a critical assessment of the model’s ability to accurately classify or predict previously unseen data. This evaluation determines the model’s effectiveness and reliability in practical applications (22). In the test set, the RBF-SVM, linear-LR, RF, XGBoost, and MLP classifiers achieved mean accuracy and mean AUC values of 97.63% and 0.987, 97.66% and 0.984, 95.01% and 0.984, 95.41% and 0.988, and 95.06% and 0.981, respectively. Considering the highest mAUC value in the test set, we selected XGBoost as the optimal classifier for our model, with average learning rate, max depth, and n_estimators values of 0.20, 3.56, and 187, respectively. Furthermore, it demonstrated a mean balanced accuracy of 93.67%, a mean sensitivity of 95.41%, and a mean specificity of 97.15%. The Friedman test revealed statistically significant differences in AUC values among the classifiers (*p* < 0.0001). Wilcoxon signed-rank test indicated that the differences between RBF-SVM and RF, RBF-SVM and MLP, RF and XGBoost, and RF and LR were statistically significant, with *p*-values <0.05 (Figure 3). The results of the permutation test confirmed the reliability of accuracy and AUC values for all models, with p-values consistently less than 0.001 in the iterations. Detailed hyperparameters for each round for all models are shown in Supplementary Table S2.

**Table 3:** The classification performance in the training set and testing set.

	Training set					Testing set				
Method	mAUC	mACC (%)	mbACC (%)	mSP(%)	mSN (%)	mAUC	mACC (%)	mbACC (%)	mSP (%)	mSN (%)
RBF-SVM	0.994	0.999 ± 0.001	0.999 ± 0.001	0.999 ± 0.037	1 ± 0	0.987	0.976 ± 0.022	0.9760.022	0.965 ± 0.037	0.987 ± 0.023
RF	0.994	0.999 ± 0	0.999 ± 0	0.999 ± 0.001	1 ± 0	0.984	0.950 ± 0.030	0.950 ± 0.030	0.931 ± 0.046	0.968 ± 0.038
Linear-LR	0.994	0.999 ± 0	0.999 ± 0.	0.999 ± 0	0.999 ± 0.001	0.984	0.976 ± 0.022	0.976 ± 0.022	0.968 ± 0.030	0.984 ± 0.029
XGBOOST	0.994	1 ± 0	1 ± 0	1 ± 0	1 ± 0	0.988	0.954 ± 0.030	0.936 ± 0.048	0.971 ± 0.039	0.954 ± 0.030
MLP	0.994	0.999 ± 0.001	0.999 ± 0.001	0.999 ± 0.001	0.999 ± 0.001	0.981	0.950 ± 0.034	0.938 ± 0.046	0.962 ± 0.045	0.950 ± 0.034

The values are expressed as mean ± standard deviation (`x ± s); AUC, area under the curve; ACC, accuracy; bACC, balance accuracy; SP, specificity; SN, sensitivity; RBF-SVM, support vector machine with a radial basis function kernel; RF, random forest; Linear-LR, logistic regression with a linear kernel; XGBoost, extreme gradient boosting; MLP, multi-layer perceptron.

### Correlation analysis

**Figure 4:**
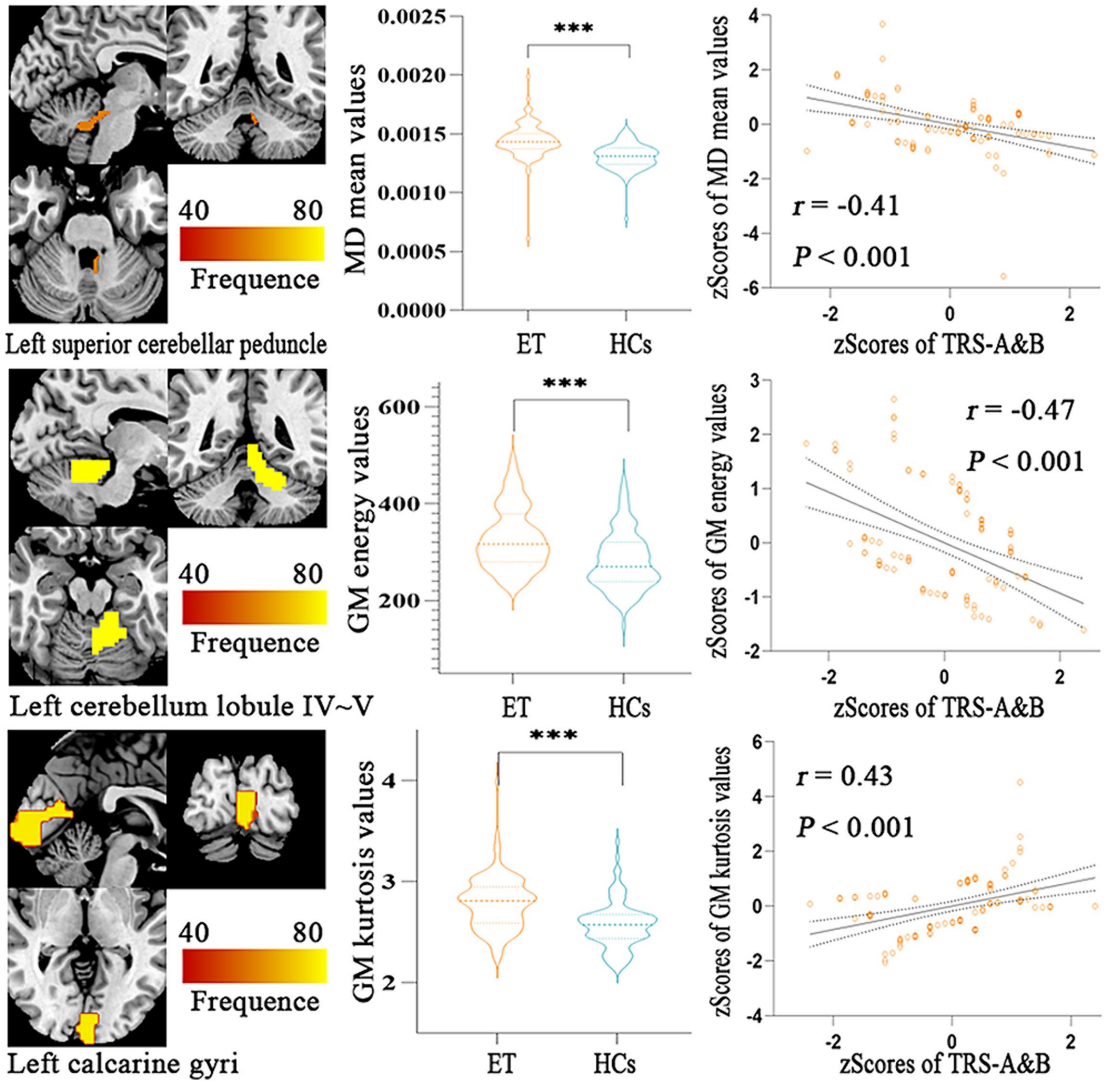
Partial Pearson Correlation analysis results between the selected radiomics features and clinical tremor characteristics in ET patients. Bonferroni multiple comparison corrections, corrected *p* < 0.05/10*(10-1)/2. Violin plots displaying the mean and standard deviations of the selected radiomics features in the ET and HCs group; Scatter plots showing the correlation analysis in the ET group. ***p < 0.001. ET, essential tremor; HCs, healthy controls; zTRS A&B scores, *z*-transformed Fahn-Tolosa-Marin Tremor Rating Scale parts A and B scores.

Figure 4 showed the partial Pearson’s correlation analysis results, and three features were significantly correlated with clinical tremor characteristics in ET patients. The mean MD in left superior cerebellar peduncle and the energy GM in left cerebellar lobule IV ~ V had a negative correlation with TRS parts A&B (*p* < 0.001, *r* = − 0.41 and - 0.47, respectively), and the kurtosis GM in left calcarine gyri had a positive correlation with TRS parts A&B (*p* < 0.001, *r* = 0.43).

## Discussion

In our study, we combined radiomics features extracted from 3D-T1 and DTI with multiple machine learning algorithms to identify ET patients from HCs and had three main findings. First, all ML algorithms (RBF-SVM, linear-LR, RF, XGBoost, and MLP classifiers) achieved excellent classification performance (with mAUC at 0.987, 0.984, 0.984, 0.988 and 0.981, respectively), and among these classifiers, XGBoost performed the best (mAUC value at 0.994). Second, the most powerful discriminative features came from both the brain GM and WM tract, and primarily located in the cerebello-thalamo-cortical (CTC) and cerebello-visual pathway. Third, some radiomics features in cerebellar GM, WM tract and visual gyri could be used to explain partially clinical tremor symptoms.

In the recent decade, due to the inherent advantages of allowing the simultaneous evaluation of multiple different source features without any *a priori* knowledge, that is, the multivariate approach, machine learning algorithms have been widely applied to identify ET (23). Using clinical characteristics such as gait and postural transition parameters (24), voice samples underwent sound signal (25), Archimedes’ spiral and wearable multi-segment upper limb tremor assessment system (26, 27), some studies have achieved good classification performance to discriminate ET from PD or ET from HCs. Meanwhile, few studies adopted MRI data as input features performed the above work, resulting in similar results. For instance, Zhang et al. employed resting-state fMRI data with SVM, Gradient Boosted Decision Tree, RF and Gaussian Naïve Bayes algorithms, achieving classification accuracies of 82.8, 79.4, 78.9, and 72.4%, respectively (28). Additionally, Jia et al. used DTI and found that the apparent diffusion coefficient (ADC) value of the red nuclei in ET patients was significantly higher compared to controls (0.90 vs. 0.77; *p* = 0.000), although no significant differences were found for FA or ADC values of other structures (29).In another study, Prasad et al. utilized 3D-T1 imaging and observed significant atrophy in the bilateral middle cerebellar peduncle, ICP, and cerebellar gray matter. Their multi-variate classifier discriminated ET from controls with a test accuracy of 86.66% (30). Compared to the above studies, our research obtained excellent classification performance, and we attributed this improvement to the following advantages: First, the diagnosis of ET patients was according to the 2018 consensus criteria in our studies, and most of the above studies just adopted the traditional consensus criteria. Almost all researchers agree that ET is a heterogeneous disease, and the heterogeneous traits cause variable and inconsistent results between different studies (31). The traditional consensus criteria paid little attention to the heterogeneity of ET, and the 2018 consensus criteria, with a more precise and narrow definition, let the cohorts of ET be more highly homogeneous. Second, 3D-T1 and DTI were used as input data, and these structural MRI images made the results more robust. The clinical characteristics and resting-state fMRI data are easily disturbed by multiple factors, such as different observers, indicators, and physiological states. However, these factors have less impact on 3D-T1 and DTI, and some metrics of 3D-T1 and DTI have been adopted as imaging markers in a lot of neurodegenerative diseases, such as Alzheimer’s disease and multiple system atrophy. Third, an AAL3 and JHU-ICBM atlas were used to comprehensively and simultaneously observe GM and WM microstructural changes, and most of the above studies only focused on GM or WM changes with tract-based spatial statistics or region of interest (ROI) methods based on some priori knowledge (32). Fourth, a large sample size (103 ET patients and 103 HCs) made our study easier to gain stable and consistent results, and except for our previous studies based on resting-state fMRI data, most of the above studies only included 20 to 40 ET patients. Therefore, we suggested that combining the radiomics features extracted from 3D-T1 and DTI with multiple machine learning algorithms would provide another important way to discriminate ET from HCs, and it would be adopted as a routine analysis in clinical practice.

The most powerful discriminative features came from both the brain GM and WM tracts, and primarily located in the cerebello-thalamo-cortical (CTC) pathway, consistent with the previous clinical, pathological and neuroimaging findings. The ventral intermediate nucleus (VIM) of the thalamus is an established therapeutic target. An increasing number of treatment methods, including stereotactic thalamotomy, deep-brain stimulus (DBS), gamma knife and focused ultrasound, have selected the VIM as the prime treatment target for ET and achieved good therapeutic effects (33–36). The VIM anatomical projects to the cerebellum and motor cortices and comprise the CTC pathway. Combined the above features give powerful evidence that the CTC pathway plays a crucial role in the generation or transmission of tremors in ET patients. Meanwhile, growing pathologic evidence is attributable to the key pathogenesis role of the cerebellum in ET. Post-mortem studies reported that loss or swelling of Purkinje cells and reducing GABA receptor density in the dentate nucleus were related to ET patients (37). Again, neuroimage from PET, structure, task, and resting-state fMRI also supported that the CTC pathway was associated with ET (38–41). However, our results were not fully consistent with the above studies. First, some radiomics of VIM, such as kurtosis MD in right ventral posterolateral (VPL) and energy MD in left ventral posterolateral (VPL), acted as the most powerful discriminative features to discriminate ET from HCs, but a correlation analysis did not explore any relationships between these features and clinical tremor status. We suggested that the VIM perhaps served as a relay station for tremor transmission from the cerebellum to the cerebral cortex in the CTC pathway and did not undertake tremor generation. Second, among the 10 most powerful discriminative features selected, 4 features, including mean MD in left inferior cerebellar peduncle (ICP), mean FA in right inferior cerebellar peduncle (ICP), mean FA in left Inferior cerebellar peduncle (ICP) and mean MD in left superior cerebellar peduncle (SCP), could be obtained by the traditional methods and the remaining 6 features existed in radiomics analysis. This profile further suggested that radiomics analysis can abstract vast quantitative features and these features also contain important information to discriminate ET from HCs.

The most powerful discriminative features located in the visual pathway seemed to be contract to most of previous studies. There is still a debate about whether the visual pathways are associated with tremors in ET patients. Using morphometric analysis of 3D-T1, most other researchers and our previous studies did not reveal any morphometric changes, including the visual areas in ET patients. However, some other studies from grip-force task fMRI reported that the visual feedback and visual areas played a vital role in modulating the severity of tremor in ET patients (42). Meanwhile, a VBM study reported that GM density changes in the visual pathway were related to ET patients (43). Again, the tremor improvement after stereotactic radio-surgical thalamotomy were involvement in the high-level visual areas (44). Finally, the most powerful discriminative features located in the visual pathway were kurtosis GM in left calcarine fissure in the present studies, and they could not be measured by the above traditional morphometric analysis methods. Therefore, we suggested that our results provide complementary information rather than contradictory information from previous studies.

### Limitations

There are several limitations that should be noted. Firstly, although the sample size of this study was relatively larger than others, it was recruited from a single center, which limited the generalizability and stability of the proposed model. Secondly, we employed strict inclusion criteria to recruit patients and had annual follow-ups. Misdiagnosing is common due to the diagnosis being strongly dependent on clinical symptoms and nervous system examinations and a lack of biomarkers in ET patients. Thirdly, the present study utilized only first-order radiomics features without considering a wide range of textural features, which are now widely used in the context of studies of neurological diseases. Additionally, some deep learning algorithms could automatically and directly extract discriminant information from the raw images. Therefore, in our future study, we hope to apply deep learning algorithms combined with MRI images to provide new insights into the microstructural changes of ET.

## Conclusion

Combined radiomics features based on 3D-T1 and DTI with multiple machine learning algorithms have achieved good classification performance for discriminating ET from HCs. The most powerful discriminatory features were not only confined to the typical tremor networks but also extended into the visual pathway, and these features would help to understand the brain microstructural pathogenesis mechanisms in ET patients.

## Data Availability

The raw data supporting the conclusions of this article will be made available by the authors, without undue reservation.

## Footnotes

^1^ http://www.fil.ion.ucl.ac.uk/spm/software/spm12

^2^ http://www.nitrc.org/projects/panda

^3^version 0.20.1, freely available: http://scikit-learn.org/.
