# Supplementary material for: Radiomics based on diffusion tensor imaging and 3D T1-weighted MRI for essential tremor diagnosis
Source: Front Neurol. 2024 Aug 27;15:1460041. doi: 10.3389/fneur.2024.1460041 (PMC11387670; doi:10.3389/fneur.2024.1460041)
Supplement: Supplementary file 1 [file Data_Sheet_1.docx]

**Radiomics Based on Diffusion Tensor Imaging and 3D T1-weighted MRI for Essential Tremor Diagnosis**

Supplementary Material

**1 Supplementary Material**

**1.1 MRI detailed scanning parameters**

DTI data were acquired using a single-shot, spin-echo, echo-planar sequence with TR/TE, 12275/86.7 ms; voxel size = 2 × 2 × 3, 30 diffusion-encoding directions with b = 1000 s/ mm2, and b = 0 s/mm2 with 8 repetitions. The acquisition of high-resolution 3D T1-W images employed the following parameters: repetition time (TR) = 8.3 ms, echo time (TE) = 3.3 ms, flip angle = 15°, slice thickness/gap = 1.0/0 mm, field of view (FOV) = 240 × 240 mm, and matrix size = 256 × 192. T2-weighted FLAIR images (TR=8,000ms, TE=126ms, TI=1,500ms, slice thickness/gap=5.0/1.5mm, FOV=240×240mm, and matrix=256×192) were also acquired.

To ensure participant comfort and minimise head motion, earplugs were used to reduce scanner noise, and foam padding was applied for head fixation. A neuroradiologist immediately inspected the images to identify any sequences affected by substantial motion artifacts, which were then re-imaged. Additionally, real-time scan images were visually assessed to exclude patients with significant vascular lesions, space-occupying lesions, or other imaging requirements that did not meet compliance standards.

**1.2 Image preprocessing steps**

The 3D T1-weighted images were preprocessed using SPM12 for Voxel-Based Morphometry (VBM) analysis. The preprocessing pipeline included several steps: first, the images were checked for any artifacts or motion-related issues. Then, they were bias-corrected to rectify intensity non-uniformities. Subsequently, the images were spatially normalised to a standard template to ensure anatomical alignment across subjects. After normalisation, the images were segmented into different tissue classes using a tissue probability map, separating white matter (WM), gray matter (GM), and cerebrospinal fluid (CSF). The resulting tissue probability maps were then normalized to the MNI space using DARTEL (Diffeomorphic Anatomical Registration Through Exponentiated Lie Algebra) normalisation.Once the normalization was completed, the resulting WM and GM segmented images were modulated. Finally, the modulated images were smoothed using an isotropic Gaussian kernel to enhance the signal-to-noise ratio and to meet the assumptions of Gaussian random field theory for statistical analysis.

Preprocessing of diffusion tensor imaging (DTI) data was performed using the PANDA toolbox version 2.2 (http://rfmri.org/DPARSF). The raw DTI data underwent standard preprocessing procedures, starting with DICOM to NIfTI conversion followed by correction for eddy current distortions and head motion artifacts.Subsequently, the preprocessed DTI images underwent brain extraction to remove non-brain tissues and were then corrected for intensity inhomogeneity. The diffusion tensor was estimated at each voxel to characterize the magnitude and directionality of water diffusion using diffusion tensor imaging fitting algorithms. From the estimated diffusion tensors, FA, MD, AD, and RD maps were derived using established formulas, providing quantitative measures indicative of tissue microstructural characteristics.

2 Supplementary Figures

**Supplementary Figure 1: Schematic overview of the nested loop classification framework**


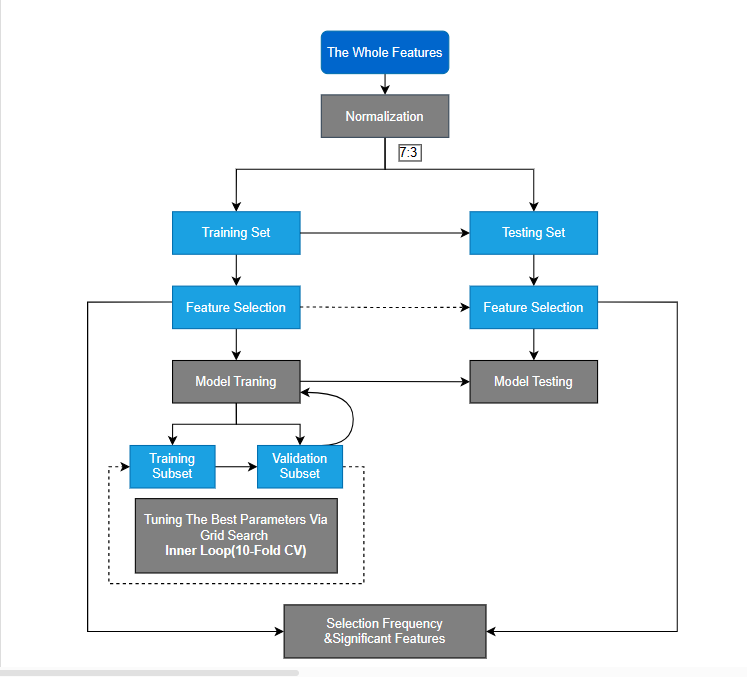


**Supplementary Figure 2:The whole radiomics analysis workflow**


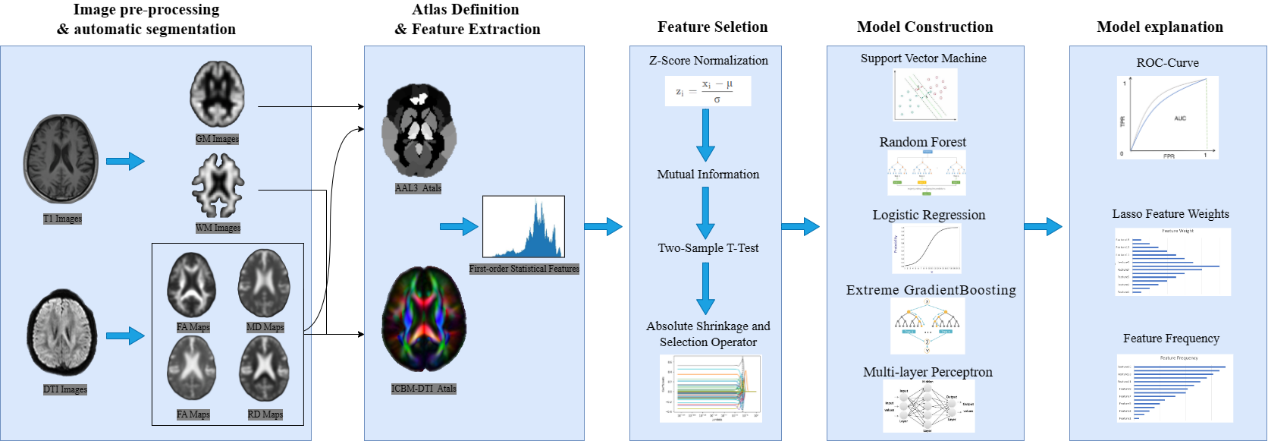


1. Supplementary table

Supplementary table 1: Detailed information on radiomics features

| Features type | Image features | Equation | Definition |
| --- | --- | --- | --- |
| First Order Features | Energy | $\sum_{\mathbf{ⅈ=1}}^{\boldsymbol{N}_{\boldsymbol{p}}} \left( \boldsymbol{X}\left( \boldsymbol{i} \right)\mathbf{+c} \right)^{\mathbf{2}}$ | Measure of the magnitude of voxel values in an image. |
|  | Total Energy | $\boldsymbol{v}_{\boldsymbol{voxel}}\sum_{\boldsymbol{r}\mathbf{=1}}^{\boldsymbol{N}_{\boldsymbol{P}}} \left( \boldsymbol{X}_{\left( \boldsymbol{i} \right)}\mathbf{+}\boldsymbol{c} \right)^{\mathbf{2}}$ | The value of Energy feature scaled by the volume of the voxel in cubic mm |
|  | Entropy | $\mathbf{-}\sum_{\boldsymbol{i}\mathbf{=1}}^{\boldsymbol{Ng}} \boldsymbol{p}\left( \boldsymbol{i} \right)\boldsymbol{log}_{\mathbf{2}} \left( \boldsymbol{p}\left( \boldsymbol{i} \right)\mathbf{+}\boldsymbol{\epsilon} \right)$ | Specifying the uncertainty/randomness in the image values |
|  | Minimum | min(X) |  |
|  | 10th percentile | The 10th percentile of X |  |
|  | 90th percentile | The 90th percentile of X |  |
|  | Maximum | max(X)maximum=max(X) | The maximum gray level intensity within the ROI |
|  | Mean | $\frac{\mathbf{1}}{\boldsymbol{N}_{\boldsymbol{P}}}\sum_{\boldsymbol{i}\mathbf{=1}}^{\boldsymbol{Np}} \boldsymbol{X}\left( \boldsymbol{i} \right)$ | The average gray level intensity within the ROI |
|  | Median |  | The median gray level intensity within the ROI |
|  | Interquartile Range | P75−P25 |  |
|  | Range | max(X)−min(X) | The range of gray values in the ROI |
|  | Mean Absolute Deviation | $\frac{\mathbf{1}}{\boldsymbol{N}}\sum_{\boldsymbol{i}\mathbf{=1}}^{\boldsymbol{N}_{\boldsymbol{p}}} \left\vert\boldsymbol{X}\left( \boldsymbol{i} \right)\mathbf{-}\bar{\boldsymbol{X}} \right\vert$ | The mean distance of all intensity values from the Mean Value of the image array |
|  | Robust Mean Absolute Deviation | $\frac{\mathbf{1}}{\boldsymbol{N}_{\mathbf{10-90}}}\sum_{\boldsymbol{i}\mathbf{=1}}^{\boldsymbol{N}_{\mathbf{10-90}}} \left\vert\boldsymbol{X}_{\mathbf{10-90}}\left( \boldsymbol{i} \right)\mathbf{-}{\bar{\boldsymbol{X}}}_{\mathbf{10-90}} \right\vert$ | The mean distance of all intensity values from the Mean Value calculated on the subset of image array with gray levels in between, or equal to the 10th and 90th percentile |
|  | Root Mean Squared | $\sqrt{\frac{\mathbf{1}}{\boldsymbol{N}_{\boldsymbol{p}}}\sum_{\boldsymbol{i}\mathbf{=1}}^{\boldsymbol{Np}} \left( \boldsymbol{X}\left( \boldsymbol{i} \right)\mathbf{+}\boldsymbol{c} \right)^{\mathbf{2}}}$ | The square-root of the mean of all the squared intensity values. Another measure of the magnitude of the image values. |
|  | Standard Deviation | $\sqrt{\frac{\mathbf{1}}{\boldsymbol{N}_{\boldsymbol{p}}}\sum_{\boldsymbol{i}\mathbf{=1}}^{\boldsymbol{Np}} \left( \boldsymbol{X}\left( \boldsymbol{i} \right)\mathbf{+}\bar{\boldsymbol{X}} \right)^{\mathbf{2}}}$ | Measuring the amount of variation or dispersion from the Mean Value |
|  | Skewness | $\frac{\frac{\mathbf{1}}{\boldsymbol{Np}}\sum_{\boldsymbol{i}\mathbf{=1}}^{\boldsymbol{N}_{\boldsymbol{P}}} \left( \boldsymbol{X}\left( \boldsymbol{i} \right)\mathbf{-}\bar{\boldsymbol{X}} \right)^{\mathbf{3}}}{\left( \sqrt{\frac{\mathbf{1}}{\boldsymbol{Np}}\sum_{\boldsymbol{i}\mathbf{=1}}^{\boldsymbol{N}_{\boldsymbol{P}}} \left( \boldsymbol{X}\left( \boldsymbol{i} \right)\mathbf{-}\bar{\boldsymbol{X}} \right)^{\mathbf{2}}} \right)^{\mathbf{3}}}$ | Measuring the asymmetry of the distribution of values about the Mean value |

Supplementary table 2: Hyperparameter search results per round of five models in machine learning methods

| SVM _gamma | LR_ parameter C | RF_number of decision trees | RF_depth of the tree | XGBoost _learning rate | XGBoost _maximum depth | XGBoost _number of decision trees | MLP _ activation function | MLP _hidden layer size | MLP _optimizer |
| --- | --- | --- | --- | --- | --- | --- | --- | --- | --- |
| 0.000488281 | 10 | 13 | $2$ | 0.5 | 3 | 200 | tanh | 256, 128, 64 | sgd |
| 0.001953125 | 10 | 24 | $6$ | 0.1 | 3 | 200 | relu | 256, 128, 64 | adam |
| 0.001953125 | 10 | 9 | $3$ | 0.5 | 3 | 200 | relu | 256, 128, 64 | adam |
| 0.001953125 | 10 | 37 | $5$ | 0.5 | 3 | 200 | relu | 256, 128, 64 | adam |
| 0.0078125 | 10 | 34 | $3$ | 0.5 | 5 | 200 | relu | 256, 128, 64 | adam |
| 0.001953125 | 10 | 29 | $4$ | 0.5 | 5 | 200 | tanh | 256, 128, 64 | sgd |
| 0.001953125 | 10 | 40 | $6$ | 0.1 | 3 | 200 | tanh | 256, 128, 64 | adam |
| 0.0078125 | 10 | 32 | $6$ | 0.1 | 3 | 200 | tanh | 256, 128, 64 | sgd |
| 0.000488281 | 10 | 14 | $2$ | 0.5 | 3 | 200 | tanh | 256, 128, 64 | adam |
| 0.00012207 | 10 | 33 | $5$ | 0.1 | 5 | 200 | tanh | 64, 128, 128, 64, 32 | adam |
| 0.001953125 | 10 | 39 | $3$ | 0.1 | 5 | 200 | tanh | 256, 128, 64 | sgd |
| 0.0078125 | 10 | 45 | $7$ | 0.5 | 5 | 200 | tanh | 64, 128, 128, 64, 32 | adam |
| 0.001953125 | 10 | 38 | $4$ | 0.2 | 5 | 200 | tanh | 64, 128, 128, 64, 32 | adam |
| 0.00012207 | 10 | 16 | $4$ | 0.1 | 3 | 200 | relu | 256, 128, 64 | adam |
| 0.000488281 | 10 | 21 | $3$ | 0.5 | 3 | 200 | relu | 256, 128, 64 | adam |
| 0.001953125 | 10 | 40 | $6$ | 0.1 | 3 | 200 | relu | 256, 128, 64 | adam |
| 0.001953125 | 10 | 15 | $3$ | 0.1 | 3 | 200 | tanh | 256, 128, 64 | sgd |
| 0.001953125 | 10 | 21 | $2$ | 0.1 | 3 | 200 | tanh | 256, 128, 64 | sgd |
| 0.000488281 | 10 | 30 | $6$ | 0.2 | 3 | 200 | relu | 256, 128, 64 | adam |
| 0.0078125 | 10 | 43 | $3$ | 0.1 | 3 | 200 | relu | 256, 128, 64 | adam |
| 0.03125 | 10 | 12 | $6$ | 0.2 | 3 | 200 | tanh | 64, 128, 128, 64, 32 | adam |
| 0.000488281 | 10 | 31 | $4$ | 0.5 | 3 | 200 | tanh | 256, 128, 64 | sgd |
| 0.000488281 | 10 | 16 | $7$ | 0.1 | 3 | 200 | tanh | 256, 128, 64 | sgd |
| 0.000488281 | 10 | 38 | $5$ | 0.5 | 3 | 200 | relu | 256, 128, 64 | adam |
| 0.000488281 | 10 | 37 | $5$ | 0.1 | 3 | 200 | relu | 256, 128, 64 | sgd |
| 0.03125 | 10 | 11 | $4$ | 0.1 | 5 | 200 | relu | 256, 128, 64 | adam |
| 0.000488281 | 10 | 38 | $6$ | 0.1 | 5 | 200 | tanh | 256, 128, 64 | adam |
| 0.001953125 | 10 | 20 | $4$ | 0.5 | 3 | 200 | tanh | 64, 128, 128, 64, 32 | adam |
| 0.001953125 | 10 | 29 | $4$ | 0.1 | 3 | 200 | tanh | 256, 128, 64 | sgd |
| 0.0078125 | 10 | 32 | $3$ | 0.2 | 5 | 200 | tanh | 256, 128, 64 | sgd |
| 0.00012207 | 10 | 24 | $5$ | 0.2 | 3 | 50 | relu | 256, 128, 64 | sgd |
| 0.0078125 | 10 | 42 | $4$ | 0.1 | 5 | 200 | tanh | 64, 128, 128, 64, 32 | adam |
| 0.0078125 | 10 | 38 | $3$ | 0.2 | 5 | 200 | tanh | 256, 128, 64 | sgd |
| 0.001953125 | 10 | 35 | $5$ | 0.1 | 5 | 200 | relu | 256, 128, 64 | adam |
| 0.001953125 | 10 | 24 | $4$ | 0.2 | 3 | 200 | tanh | 256, 128, 64 | sgd |
| 0.0078125 | 10 | 38 | $3$ | 0.2 | 5 | 200 | relu | 256, 128, 64 | adam |
| 0.00012207 | 10 | 47 | $4$ | 0.2 | 3 | 200 | tanh | 256, 128, 64 | adam |
| 0.03125 | 10 | 24 | $5$ | 0.1 | 5 | 200 | relu | 256, 128, 64 | adam |
| 0.000488281 | 10 | 16 | $5$ | 0.2 | 3 | 200 | tanh | 256, 128, 64 | adam |
| 0.0078125 | 10 | 23 | $4$ | 0.5 | 3 | 200 | relu | 256, 128, 64 | adam |
| 0.001953125 | 10 | 38 | $6$ | 0.2 | 3 | 50 | tanh | 256, 128, 64 | sgd |
| 0.001953125 | 10 | 17 | $4$ | 0.1 | 3 | 50 | tanh | 64, 128, 128, 64, 32 | sgd |
| 0.0078125 | 10 | 21 | $3$ | 0.2 | 3 | 200 | tanh | 256, 128, 64 | adam |
| 0.000488281 | 10 | 27 | $3$ | 0.1 | 5 | 200 | relu | 256, 128, 64 | adam |
| 0.001953125 | 10 | 22 | $4$ | 0.2 | 5 | 200 | tanh | 64, 128, 128, 64, 32 | adam |
| 0.00012207 | 10 | 40 | $5$ | 0.2 | 3 | 50 | relu | 256, 128, 64 | adam |
| 0.000488281 | 10 | 21 | $4$ | 0.5 | 3 | 200 | relu | 256, 128, 64 | adam |
| 0.001953125 | 10 | 22 | $2$ | 0.5 | 3 | 200 | tanh | 256, 128, 64 | sgd |
| 0.001953125 | 10 | 36 | $4$ | 0.1 | 3 | 200 | tanh | 256, 128, 64 | sgd |
| 0.001953125 | 10 | 48 | $4$ | 0.1 | 3 | 200 | tanh | 256, 128, 64 | adam |
| 0.000488281 | 10 | 40 | $3$ | 0.2 | 3 | 200 | tanh | 256, 128, 64 | adam |
| 0.0078125 | 10 | 28 | $5$ | 0.1 | 3 | 200 | tanh | 256, 128, 64 | adam |
| 0.001953125 | 10 | 22 | $5$ | 0.1 | 3 | 200 | tanh | 256, 128, 64 | adam |
| 0.001953125 | 10 | 17 | $4$ | 0.1 | 3 | 200 | relu | 256, 128, 64 | sgd |
| 0.0078125 | 10 | 43 | $4$ | 0.5 | 5 | 200 | tanh | 256, 128, 64 | sgd |
| 0.0078125 | 10 | 24 | $4$ | 0.1 | 3 | 200 | relu | 64, 128, 128, 64, 32 | adam |
| 0.001953125 | 10 | 44 | $7$ | 0.5 | 5 | 200 | relu | 256, 128, 64 | adam |
| 0.00012207 | 10 | 15 | $6$ | 0.2 | 5 | 200 | relu | 256, 128, 64 | adam |
| 0.000488281 | 100 | 18 | $4$ | 0.1 | 3 | 200 | tanh | 256, 128, 64 | sgd |
| 0.00012207 | 10 | 19 | $5$ | 0.1 | 3 | 200 | tanh | 256, 128, 64 | sgd |
| 0.0078125 | 10 | 19 | $2$ | 0.2 | 5 | 200 | tanh | 256, 128, 64 | sgd |
| 0.001953125 | 10 | 43 | $4$ | 0.1 | 3 | 100 | tanh | 256, 128, 64 | adam |
| 0.000488281 | 10 | 34 | $6$ | 0.1 | 3 | 200 | tanh | 256, 128, 64 | adam |
| 0.00012207 | 10 | 45 | $5$ | 0.2 | 3 | 200 | tanh | 256, 128, 64 | adam |
| 0.000488281 | 10 | 31 | $4$ | 0.2 | 5 | 200 | tanh | 256, 128, 64 | sgd |
| 0.000488281 | 10 | 36 | $5$ | 0.1 | 3 | 50 | tanh | 256, 128, 64 | sgd |
| 0.001953125 | 10 | 25 | $5$ | 0.1 | 3 | 200 | relu | 256, 128, 64 | adam |
| 0.000488281 | 10 | 18 | $6$ | 0.2 | 3 | 200 | relu | 256, 128, 64 | adam |
| 0.001953125 | 10 | 29 | $5$ | 0.2 | 5 | 200 | tanh | 256, 128, 64 | sgd |
| 0.000488281 | 10 | 46 | $6$ | 0.1 | 3 | 200 | tanh | 256, 128, 64 | sgd |
| 0.03125 | 10 | 46 | $7$ | 0.5 | 3 | 200 | tanh | 256, 128, 64 | adam |
| 0.001953125 | 10 | 46 | $6$ | 0.1 | 5 | 200 | tanh | 256, 128, 64 | sgd |
| 0.001953125 | 100 | 33 | $4$ | 0.1 | 3 | 50 | tanh | 256, 128, 64 | adam |
| 0.000488281 | 10 | 36 | $6$ | 0.5 | 5 | 200 | tanh | 64, 128, 128, 64, 32 | adam |
| 0.000488281 | 10 | 14 | $3$ | 0.2 | 3 | 200 | tanh | 256, 128, 64 | adam |
| 0.001953125 | 10 | 46 | $5$ | 0.2 | 3 | 200 | tanh | 256, 128, 64 | sgd |
| 0.000488281 | 10 | 40 | $3$ | 0.1 | 3 | 200 | tanh | 64, 128, 128, 64, 32 | adam |
| 0.000488281 | 10 | 38 | $3$ | 0.5 | 3 | 200 | tanh | 256, 128, 64 | adam |
| 0.001953125 | 10 | 21 | $3$ | 0.1 | 3 | 200 | tanh | 64, 128, 128, 64, 32 | sgd |
| 0.001953125 | 10 | 39 | $4$ | 0.2 | 5 | 200 | tanh | 256, 128, 64 | sgd |
| 0.001953125 | 10 | 16 | $5$ | 0.1 | 3 | 200 | relu | 256, 128, 64 | adam |
| 0.001953125 | 10 | 43 | 5 | 0.1 | 3 | 200 | relu | 256, 128, 64 | sgd |
| 0.001953125 | 10 | 23 | 4 | 0.5 | 3 | 200 | tanh | 256, 128, 64 | sgd |
| 0.001953125 | 10 | 28 | 3 | 0.5 | 3 | 200 | tanh | 256, 128, 64 | adam |
| 0.001953125 | 10 | 14 | 6 | 0.2 | 3 | 200 | tanh | 64, 128, 128, 64, 32 | sgd |
| 0.000488281 | 10 | 42 | 4 | 0.5 | 3 | 200 | tanh | 256, 128, 64 | sgd |
| 0.001953125 | 10 | 48 | 5 | 0.1 | 3 | 200 | tanh | 256, 128, 64 | adam |
| 3.05176E-05 | 10 | 30 | 2 | 0.1 | 3 | 200 | tanh | 256, 128, 64 | sgd |
| 0.001953125 | 10 | 34 | 6 | 0.1 | 3 | 200 | tanh | 256, 128, 64 | sgd |
| 0.001953125 | 10 | 33 | 3 | 0.1 | 5 | 50 | relu | 64, 128, 128, 64, 32 | adam |
| 0.001953125 | 10 | 30 | 4 | 0.5 | 3 | 200 | tanh | 256, 128, 64 | sgd |
| 0.0078125 | 100 | 17 | 6 | 0.5 | 3 | 200 | tanh | 256, 128, 64 | sgd |
| 0.001953125 | 10 | 14 | 4 | 0.5 | 3 | 200 | tanh | 256, 128, 64 | sgd |
| 0.001953125 | 10 | 16 | 4 | 0.5 | 5 | 200 | tanh | 256, 128, 64 | adam |
| 0.001953125 | 10 | 26 | 2 | 0.1 | 3 | 200 | relu | 256, 128, 64 | sgd |
| 0.001953125 | 10 | 17 | 6 | 0.2 | 5 | 200 | tanh | 256, 128, 64 | sgd |
| 0.000488281 | 10 | 35 | 4 | 0.1 | 3 | 200 | tanh | 64, 128, 128, 64, 32 | adam |
| 0.001953125 | 10 | 40 | 3 | 0.1 | 3 | 200 | tanh | 256, 128, 64 | adam |
| 0.000488281 | 10 | 49 | 3 | 0.1 | 3 | 200 | tanh | 256, 128, 64 | adam |
| 0.001953125 | 10 | 46 | 5 | 0.1 | 3 | 50 | relu | 256, 128, 64 | adam |

SVM:support vector machine; 6LR:logistic regression; RF:random forest; XGBoost:extreme gradient boos3ting; MLP:multi-layer perceptron.
